# Nanotechnology for Environmental and Biomedical Research

**DOI:** 10.3390/nano10112220

**Published:** 2020-11-08

**Authors:** Giada Frenzilli

**Affiliations:** Department of Clinical and Experimental Medicine, University of Pisa, 56126 Pisa, Italy; giada@biomed.unipi.it; Tel.: +39-050-2219111

Given the high production and broad feasibility of nanomaterials, the application of nanotechnology includes the use of engineered nanomaterials (ENMs) to clean-up polluted media such as soils, water, air, groundwater and wastewaters, and is known as nanoremediation. Contamination by hazardous substances in environmental matrices, including landfills, oil fields, and manufacturing and industrial sites, represents a global concern and needs to be remediated, since it poses a serious risk for the environment and human health. Particular attention should also be focused on the use of medical devices and recent developments in the use of nanoparticles expressed as drug delivery systems designed to treat a wide variety of diseases. This Special Issue, “Nanotechnology for Environmental and Biomedical Research”, is characterized by this double role, and is aimed at facing the use of nanotechnology for environmental and human health. It aims at collecting a compilation of articles that strongly demonstrate the continuous efforts in developing advanced and safe nanomaterial-based technologies for nanoremediation, as well as for drug delivery and other biomedical applications. The present Special Issue has covered the most recent advances in the safe nanomaterials synthesis field, as well as in environmental applications, the use of restorative materials, drug delivery, and other clinical applications. In this Special Issue, thirteen selected original research papers and three reviews are collected. More than 80 scientists from universities and research institutions contributed through their research studies and expertise for the success of this Special Issue. The scientific contributions are summarized in the next paragraphs. Ten contributions, including two reviews, are related to environmental applications. Riva and co-authors [1] proposed a nanostructured cellulose-based material as a possible sorbent for the removal of organic dyes from water, demonstrating its sorbent efficiency for four different organic dyes commonly used for fabric printing. The material performance was compared with that of an activated carbon, routinely used for this application, thus highlighting the potentialities and limits of this new material together with the important issue of the regeneration and reuse of the sorbent. The suitability of cellulose-based nanomaterials for the remediation of heavy metal-contaminated environments was then assessed by Guidi and colleagues [2]. They indicated an eco-friendly cellulose-based nanosponge as a safe and effective candidate in the cadmium remediation process, being able to sequester cadmium and restore cellular damage induced by cadmium exposure in the zebra mussel, an animal model typical of freshwater environments, without altering cellular physiological activity. In particular, the authors showed the recovery of cadmium-induced DNA integrity loss, cell proliferation increase, and nuclear morphology and chromosomal alterations in zebra mussel haemocytes. The same ecofriendly nanosponges were demonstrated to be effective in the removal of zinc ions from the seawater environment, through another in vivo study. The contribution by Liberatori et al. [3] confirmed, besides the efficacy of the nanosponge, the recovery of the toxicological responses induced in the marine mussel. The genetic, chromosomal, cellular and tissue alterations induced by zinc ions were actually reported at control levels, supporting the environmentally safe application of cellulose-based nanosponges for heavy metal removal from seawater. Mariano and colleagues [4] demonstrated the possible use of a microalgae as a model microorganism to study silver nanoparticle toxicity, but also to protect against nanoparticle pollution. They showed that silver nanoparticles, internalized in a time- and dose-dependent manner inside large vacuoles, were not released into the medium for almost one week, without undergoing any biotransformation, confirming the role of the microalgae in environmental protection. Still within the field of remediation, Park and co-authors [5] proposed a nanohybrid material able to detect uranyl ions spectroscopically and act as a uranyl ion absorbent in an aqueous system. The contribution has high impact because the uranyl ion, the most soluble toxic uranium species, is considered as an important index for monitoring nuclear wastewater quality. Furthermore, pitch-based activated carbon fibers, prepared by steam activation, were proposed by Lee and colleagues [6] as a solution for unburned hydrocarbon car emission removal. The pitch-based activated carbon fibers actually exhibited enhanced butane working capacity and adsorption velocity when compared to commercial products, with lower concentrations of n-butane due to their characteristic pore structure. Besides nanostructured materials, nanotoxicology and nanoremediation, another keyword was Trojan horse effect. Two contributions faced this research aspect, which concerns the interactions between nanostructured materials and classical pollutants. The assessment of the ecotoxicological effects of the interaction between benzo[a]pyrene and fullerene (C60) was performed by Barranger and co-authors [7] in the marine mussel. They found antagonistic responses at the genotoxic and proteomic level, also showing a complex multi-modal response to environmental stressors in the species used. Another antagonistic interaction was reported by Santonastaso and co-workers [8], who assessed the in vitro effects of titanium dioxide nanoparticles and cadmium interaction in human sperm cells by investigating semen parameters, apoptotic processes, DNA integrity, genomic stability and oxidative stress. They demonstrated that the genotoxicity induced by the co-exposure was lower if compared to single cadmium exposure, suggesting the formation of a sandwich-like structure, with cadmium in the middle, to explain the inhibition of its genotoxicity in human sperms. In order to conclude the section concerning the environmental aspects of nanotechnology research, two valuable reviews are reported here. The one by Boros and Ostafe [9] reviewed the ecotoxicological effects of nanomaterials as well as their testing methods, which are adaptable for nanomaterials. The authors reported a broad spectrum of genetic, molecular, cellular, morphological and behavioral effects, involving a wide range of organisms, such as algae, duckweed, amphipods, daphnids, chironomids, terrestrial plants, nematodes and earthworms. It is interesting to note that, having reported values mainly for aquatic ecotoxicity, the most sensitive test turned out to be the algae assay, and the most toxic nanomaterials were composed of silver, reinforcing the impact of the contribution by Mariano et al. [4]. The other review, by Zahra et al. [10], mainly focused on the aspect of environmental safety, and gave an overview of the potential exposure route of titanium dioxide nanoparticles from industrial applications to wastewater treatment, and the impact of this on the agro-environment. The increasing interest in the role of nanotechnology in nanosafe applications is paralleled in the scientific arena by the extreme need to upgrade our knowledge via the use of nanotechnology in the human health field. In this context, this Special Issue collects five research articles and one review covering the principal aspects of nanotechnology applied to biomedical applications, such as the study of nanoparticles used as antibiotics and restorative material, as well as nanomaterials able to deliver drugs or to modulate stem cell trafficking. The original contribution by the group of Tsakmakidis [11] investigated the effect of Fe_3_O_4_ nanoparticles on an animal model to assess their ability to substitute antibiotics additives in extending semen storage time. The authors found a significant reduction in the bacterial load in the samples incubated with Fe_3_O_4_ nanoparticles in comparison with controls after both 24 and 48 hours. Moreover, no adverse effects on sperm characteristics, such as morphology, viability or DNA integrity, were detected, offering important information concerning semen handling in the artificial insemination field. A novel method of preparing reduced graphene oxide from graphene oxide was developed, employing a vegetable extract, by Uman and co-authors [12]. They showed that the “green” modification of graphene oxide leads to an enhancement in antibacterial activity against Gram-positive and Gram-negative bacteria, besides increasing antibiofilm activity on a human breast cancer cell line, thus indicating reduced graphene oxide nanoparticles as a potential anticancer agent. Another biomedical application was proposed by the group of Genaro and colleagues [13], who indicated the incorporation of nanohydroxyapatite in resin-modified glass ionomer cement, a restorative material, as a method to increase the cell viability and biocompatibility performance of odontoblastoid cells. The biophysical effect of nanomaterials within stem cell-based therapies has been investigated by Shin and co-authors [14]. They labeled human bone marrow-derived mesenchymal stem cells with silica-coated magnetic nanoparticles incorporating rhodamine B isothiocyanate, and found decreased cell viability, an increase in intracellular reactive oxygen species, and, most of all, a decrease in stem cell migration activity related with membrane fluidity reduction and focal adhesion impairment. Therefore, the authors highlighted the importance of nanoparticles that are used for stem cell trafficking or clinical applications being labeled using optimal nanoparticle concentrations, so as to preserve human bone marrow-derived mesenchymal stem cells’ migratory activity, thus ensuring successful outcomes following stem cell localization. Regarding another key word of the Special Issue, i.e., drug delivery, an interesting contribution came from Matsuo and co-authors [15], who indicated encapsulated lipid-based nanoparticles, prepared from neutral hydrogenated soybean phosphatidylcholine and dipalmitoylphosphatidylglycerol, as an optimal way, after roll grinding and high-pressure homogenization, to prepare stable bicelles for nifedipine delivery. Cryo-transmission electron microscopy and atomic force microscopy were also performed to better understand the structure of such nifedipine-encapsulated lipid-based nanoparticles. So, taking into consideration the result of long term stability, standard nifedipine-encapsulated lipid-based nanoparticle bicelles (5 liposomes/1 micelles) showed the most long-term stabilities, illustrating a useful preparation method for stable bicelles to be employed in the drug delivery field. A review of the role of nanoemulsions in cancer therapeutics [16] effectively concludes this excursus on nanotechnology and biomedical applications. Nanoemulsions are pharmaceutical formulations, made of particles within the nanometric range, which are able to encapsulate drugs that are poorly hydrophilic due to their hydrophobic core. Sánchez-López and co-workers reviewed the characteristics of nanoemulsions that face and overcome problems such as water solubility, targeting specificity and multidrug resistance. Nanoemulsions can actually be modified by the use of ligands of different natures to target specific tumor cell components or to avoid multidrug resistance. A broad spectrum of methodologies through which nanoemulsions can be designed to achieve successful therapeutic outcomes in several types of cancer are reported. Applications of nanoemulsions in colon, ovarian, prostate, breast, lung, leukemia and melanoma cancer therapy, as well as in nanotheragnostics and drug delivery, have been widely discussed. In conclusion, the papers collected in this Special Issue cover the most relevant advanced applications of nanotechnology in the environmental and human health fields, also providing new research directions to be expanded in the near future.
